# Use of structural models to elucidate the occurrence of falls among older adults according to abdominal obesity: a cross-sectional study

**DOI:** 10.1590/1516-3180.2021.0738.R1.07042022

**Published:** 2022-09-12

**Authors:** Elma Lúcia de Freitas Monteiro, Érica Midori Ikegami, Nayara Gomes Nunes Oliveira, Erika Cardoso dos Reis, Jair Sindra Virtuoso

**Affiliations:** I ^I^MSc. Nutritionist and PhD Student, Postgraduate Program in Health Care, Universidade Federal do Triângulo Mineiro (UFTM), Uberaba (MG), Brazil.; II ^II^MSc. Physiotherapist and PhD Student, Program in Health Care, Universidade Federal do Triângulo Mineiro (UFTM), Uberaba (MG), Brazil.; III ^III^MSc, PhD. Nurse specialist in the health of older adults, Clinical Hospital, Universidade Federal de Uberlândia (UFU), Uberlândia (MG), Brazil.; IV ^IV^MSc, PhD. Nutritionist, Associate Professor, Department of Clinical and Social Nutrition, Universidade Federal de Ouro Preto (UFOP), Ouro Preto (MG), Brazil.; V ^V^MSc, PhD. Physical Education Professional and Associate Professor II, Department of Sports Science, Universidade Federal do Triângulo Mineiro (UFTM), Uberaba (MG), Brazil.

**Keywords:** Aged, Accidental falls, Obesity, abdominal, Models, statistical, Aging, Multimorbidity, Elderly, Falls, Abdominal obesity, Path analysis

## Abstract

**BACKGROUND:**

Obesity is a risk factor for falls in older adults, but the effects of body fat distribution and its interaction with other factors are not well established.

**OBJECTIVES:**

To verify the occurrence of falls among older adults with and without abdominal obesity and the effects of sociodemographic, health, and behavioral variables on this outcome.

**DESIGN AND SETTING:**

A cross-sectional study in an urban area of Alcobaça, Brazil.

**METHODS:**

Men and women older than 60 years with (270) and without (184) abdominal obesity were included. Sociodemographic, health, and behavioral data were collected using validated questionnaires in Brazil. Descriptive and path analyses were performed (P < 0.05).

**RESULTS:**

The occurrence of falls was high in participants with abdominal obesity (33.0%). In both groups, a higher number of morbidities (β = 0.25, P < 0.001; β = 0.26, P = 0.002) was directly associated with a higher occurrence of falls. Among participants without abdominal obesity, a lower number of medications (β = -0.16; P = 0.04), a higher number of depressive symptoms (β = 0.15; P = 0.04), worse performance on the agility and dynamic balance tests (β = 0.37; P < 0.001), and lower functional disability for basic activities of daily living (β = -0.21; P = 0.006) were directly associated with the occurrence of falls.

**CONCLUSION:**

Adults older than 60 years with abdominal obesity have a higher prevalence of falls. Different factors were associated with the occurrence of falls in both groups.

## INTRODUCTION

Falls are a public health problem at the global level due to the associated repercussions, such as a large number of years of disability, the need for long-term care, and high mortality rates.^1^ In the Brazilian population there is a prevalence of 27% for falls among older adults, with advanced age among the risk factors with strong scientific evidence for falls.^2,3^

Falls have also been associated with the presence of morbidities,^4,5^ depressive symptoms,^3,6^ changes in balance and reduction in muscle strength,^7–9^ use of medicines,^10,11^ disabilities in basic activities (BADL) and instrumental activities (IADL) of daily living,^12,13^ lower levels of physical activity,^14^ prolonged sedentary behavior,^15^ female sex^2,3^ and obesity.^16–18^

Among these factors, obesity, which is also currently a public health problem, requires further investigation. However, the effects of body fat distribution on the occurrence of falls are not yet well established.^18^ The scientific literature shows that excess fat in the central region contributes to the anterior displacement of the body’s center of mass, making it difficult to stabilize in an upright posture, shifting the line of gravity that approaches the body’s base of support.^19^

Based on studies in older adults, it appears that falls are associated with sociodemographic, health, and behavioral characteristics.^2–18^ However, which of these factors act directly or indirectly to mediate the occurrence of falls in older adults with and without abdominal obesity remains unclear.

Obesity is a common condition in the elderly population and is associated with higher morbidity and mortality, risk of institutionalization, and poorer quality of life.^20^ Additionally, advanced age is one of the main factors associated with falls.^1^ Thus, it is necessary to expand on the understanding of the association between obesity and falls in this population, aiming to identify individuals at greater risk and propose preventive measures.^18^

For a better understanding of the event, analyses with structural equation models are necessary to allow for the simultaneous identification of the dependence and interrelation of multiple variables. Moreover, it is necessary to estimate the direct and mediated effects by other factors that may integrate into the causal network of the result of interest.^21^

This type of analysis has not been explored in this context. It can expand on the knowledge of falls in older adults with and without abdominal obesity, and provide support to the need for elaboration of actions aimed at improving the health care in this population.

## OBJECTIVE

This study aimed to determine the occurrence of falls among older adults with and without abdominal obesity and the direct and indirect effects of sociodemographic, health, and behavioral variables on this outcome.

## METHODS

### Study design and setting

We used a quantitative approach with a cross-sectional design. It was conducted as part of the Brazilian project titled “Longitudinal Study of the Health of Older Adults in Alcobaça, Bahia” (ELSIA). The Program to Strengthen the Report of Observational Studies in Epidemiology (STROBE) guided the research report.

Data collection was carried out from July to October 2015 in the houses of adults aged greater than 60 years and consisted of the application of questionnaires, physical performance tests, and verification of anthropometric measurements. The selected interviewers underwent training, qualifications, and ethical research procedures.

The project was approved by the Ethics Committee for Research with Human Beings under protocol no. 966.983 on February 27, 2015. The interviewers contacted the participants at home and presented the research objectives and the free and informed consent form. The interviews started once participants signed the consent and terms forms.

### Participants and eligibility criteria

Men and women aged 60 years or older registered in the Family Health Strategy and living in the urban area of the municipality of Alcobaça, state of Bahia participated in the study. In 2015, the municipality had a total of 21,319 inhabitants, of which 2,047 were aged 60 years or older and 1,024 lived in urban areas^22^, with 743 registered in the Family Health Strategy. The municipality had all residences assisted by the Family Health Strategy in 2015;^23^ however, in a survey conducted by community health agents, data from 743 older adults were made available.

The exclusion criteria for participants were: bedridden or hospitalized during the study period; residents’ of long-term care institutions; severe visual and auditory acuity difficulties which could make communication with the interviewer difficult; wheelchair dependent; musculoskeletal or neurological diseases that prevented the performance of physical tests and anthropometric measurements; and cognitive decline (evaluated according to the Mini-Mental State Examination, in which a cutoff point of ≤ 12 points was used, regardless of education level, due to the high illiteracy rate among older adults in the municipality).^24^

Of the 743 eligible participants, six were wheelchair users, 10 were bedridden, 19 had diseases that made it impossible to carry out the interview, 14 had cognitive decline; eight had communication difficulties, and one had conditions that prevented communication during the interview. Moreover, 54 refused to participate, 158 were not at home in the interviewer’s three attempts, and 19 did not complete the full interview. Thus, 454 older adults comprised the study sample, divided into two groups: those with abdominal obesity (270) and those without abdominal obesity (184).

### Data collection

#### Waist circumference

With the participant in an orthostatic position and wearing as little clothing as possible, waist circumference was measured at the midpoint between the last rib and the iliac crest with a flexible and inelastic tape during normal expiration. Cutoff values of ≤ 102 cm for men and ≤ 88 cm for women were used to classify participants into the “without abdominal obesity” group and > 102 cm for men and > 88 cm for women into the “with abdominal obesity” group.^25^

### Occurrence of falls (dependent variable)

The occurrence and number of falls in the last 12 months were determined with questions widely used in gerontological research:^5,17^ (1) “Have you had any falls in the last 12 months?” (2) “If so, how many times?” The dependent variables in the present study were the report of falls (yes or no) and the average number of falls.

### Independent variables

Sociodemographic and economic data, continuous use of medications, and the presence of morbidities were obtained through the application of a structured questionnaire constructed by the researchers.

Sociodemographic and economic data were: sex (female; male); age group (60–69; 70–79; 80 or more) and age in complete years (average), marital status (single; married; widowed; divorced), self-reported skin color/race (white; black; brown; indigenous), years of completed education (none; 1–4, 5 or more), housing arrangement (lives alone; lives with someone) and economic class (A-B; C; D-E). Regarding the use of medications and the presence of morbidities, participants were asked if they used them continuously (yes or no) and if they had any of the diseases included in a list prepared by the researchers (yes; no). The average number of morbidities and continuous medications were used for calculations.

The presence of depressive symptoms was determined using the validated Brazilian version of the Abbreviated Geriatric Depression Scale, which provides a score ranging from 0 to 15 points.^26^ The average number of depressive symptoms was considered.

Regarding functional capacity, BADL was evaluated using the Katz Index, which ranges from 0 to 6 points.^27^ For IADL, the Lawton and Brody Scale was used, which ranges from 7 (highest level of dependence) to 21 points (complete independence).^28^ Both instruments are adapted to reflect the Brazilian population and the scores in each of the scales were considered, with higher scores for BADL and lower scores for IADL indicating higher functional disability.

The level of physical activity was determined using the long version of the International Physical Activity Questionnaire (IPAQ), which has been adapted for older adults.^29^ For the main analysis, participants were categorized, according to active time as insufficiently active (< 150 minutes/week) or sufficiently active (≥ 150 minutes/week).^30^

Sedentary behavior was obtained through two IPAQ questions, determined according to the total sitting time, in minutes per day, using the weighted average of time spent sitting on one day of the week and on one day of the weekend.^31^

Agility and dynamic balance were measured by the time spent performing the “Timed Up and Go” test, a modified version of the Fullerton test battery proposed by Rikli and Jones.^32^ The variable was measured according to the time in seconds, in quantitative mode.

Handgrip strength was measured using a SAEHAN dynamometer (SH5001, Korea) with individual adjustment according to hand size and measurement performed according to Dias et al.^33^ The variable was provided in kilogram-force (kgf), in quantitative mode.

### Data analysis

Statistical analyses were performed using IBM Corp. Statistical Package for Social Sciences (SPSS, version 24.0; IBM Corp). Analysis of Moment Structures (AMOS), version 24.0 (Armonk, New York, United States). Data were subjected to descriptive analysis using absolute and relative frequencies for categorical variables and means and standard deviations for quantitative variables.

**Figure 1.**
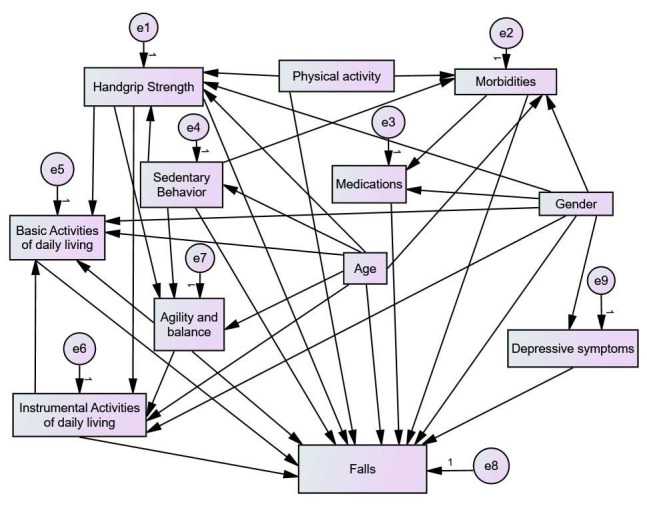
The initial model proposed for the analysis of association between sociodemographic, health, and behavioral variables and the occurrence of falls among older adults according to abdominal obesity, Alcobaça, Bahia, Brazil, 2021.

Following this, structural equation modeling was carried out through path analysis, which allowed the simultaneous verification of the dependence and interrelationship of multiple variables, in addition to estimating the direct and mediated effects of other factors that integrate into the causal network of the outcome of interest.^21^ In building the model, it was considered that sociodemographic, health, and behavioral characteristics are associated with falls through direct and indirect trajectories. In this scenario, a hypothetical model was developed (Figure 1), tested through path analysis,^21^ composed of observed variables, represented by rectangles, and classified as endogenous and exogenous. Endogenous variables receive directional arrows and measurement errors are attributed, as specified by the letter “e” in the models.^21^

From the specified hypothetical model (Figure 1), three steps for the analysis of structural equation modeling were carried out: data collection, model estimation, and assessment of the adequation of fit.^21^ The parameters were estimated using the free asymptotic distribution method, and the fit qualities of the models were evaluated according to the Chi-square test (χ^2^) P > 0.05; goodness of fit index (GFI) ≥ 0.95; comparative fit index (CFI) ≥ 0.95; Tucker-Lewis Index (TLI) ≥ 0.90; and root mean error of approximation (RMSEA) ≤ 0.05.^21^ The hypothetical model was tested, and the adjustments were carried out later. For this purpose, non-significant pathways were eliminated (P > 0.05), and modification indices (≥ 11) were calculated.^21^

In the path analysis, the variables of age, morbidities, use of continuous medications, depressive symptoms, functional capacity for BADL and IADL, agility and balance, hand grip strength, sedentary behavior, and falls were used quantitatively, considering for completed years of life; number of morbidities, medications, and depressive symptoms; scores of activities of daily living (BADL/IADL); agility and balance; hand grip strength; sedentary behavior; and number of falls.

Direct associations were presented through estimates of standardized coefficients in the trajectories between sociodemographic, health, and behavioral variables and falls. Indirect effects (mediation effects) were determined from the intermediate trajectories of the aforementioned variables. In all tests, type I error was set at 5% (P value < 0.05).

## RESULTS

The participants (n = 454) were divided into two groups: those with abdominal obesity (n = 270) and those without (n = 184). In both groups, those aged 60–69 years, married, black, with 1-4 years of education, who lived with someone, and who were sufficiently active (Table 1) predominated. Regarding sex and economic class, most participants in the group with abdominal obesity were women and economic class C, and most participants in the group without abdominal obesity were men and classes D-E (Table 1).

**Table 1.** Frequency distribution for sociodemographic and economic characteristics and the physical activity practices among older adults according to abdominal obesity, Alcobaça, Bahia, Brazil, 2021

Variables	Without abdominal obesity		With abdominal obesity	
	n	%	n	%
**Gender**				
Female	66	35.9	217	80.4
Male	118	64.1	53	19.6
**Age group**				
60–69	104	56.5	149	55.2
70–79	52	28.3	82	30.4
80 or over	28	15.2	39	14.4
**Marital status**				
Single	18	9.8	23	8.5
Married	107	58.2	106	39.3
Widowed	29	15.8	92	34.1
Divorced	30	16.3	49	18.1
**Race/ethnicity**				
White	53	28.8	92	34.1
Black	71	38.6	93	34.4
Brown	60	32.6	83	30.7
Indigenous	0	0	2	0.7
**Years of study**				
None	59	32.1	88	32.6
1–4	70	38.0	93	34.4
5 or over	55	29.9	89	33.0
**Housing arrangement**				
Alone	28	15.2	45	16.7
Accompanied	156	84.8	225	83.3
**Economic class**				
A-B	25	13.6	41	15.2
C	75	40.8	116	43.0
D-E	84	45.7	113	41.9
**Physical activity**				
Insufficiently active	104	56.5	139	51.5
Sufficiently active	80	43.5	131	48.5

Table 2 lists the means and standard deviations of the variables included in the tested model. Only sex and physical activity were considered dichotomous and inserted into the model according to the presence of abdominal obesity, as shown in Table 1.

**Table 2.** Means and standard deviations of sociodemographic, health and behavioral variables included in the model among older adults according to abdominal obesity, Alcobaça, Bahia, Brazil, 2021

Variables	Without abdominal obesity	With abdominal obesity
	Mean (±)	Mean (±)
Age (complete years)	70.26 (8.54)	69.96 (7.89)
Agility and dynamic balance (time in seconds)	6.28 (3.55)	7.14 (5.08)
Medications (number)	2.02 (2.03)	2.83 (2.30)
Sedentary behavior time (min/day)	415.4 (162.9)	436.3 (154.5)
BADL (0 to 12 scale)	0.32 (0.87)	0.28 (0.58)
IADL (7 to 14 scale)	11.52 (2.85)	11.41 (2.70)
Number of depressive symptoms (0 to 15 scale)	2.15 (2.12)	3.00 (2.81)
Handgrip strength (kgf)	26.35 (9.16)	21.86 (7.52)
Morbidities (number)	4.70 (3.93)	6.64 (4.79)
Falls in the last 12 months (number)	0.33 (0.76)	0.72 (1.52)

± = standard deviation; min = minutes; BADL = basic activities of daily living; IADL = instrumental activities of daily living; kgf = kilogram-force.

The prevalence of falls in the study population was 28.6%. The percentage of falls in the last 12 months was 33.0% in the group with abdominal obesity, and 22.3% in the group without abdominal obesity. Further, participants with abdominal obesity were more likely to fall than those without obesity (odds ratio [OR] = 1.71, confidence interval, CI = 1.12–2.74; P = 0.013).

**Figure 2.**
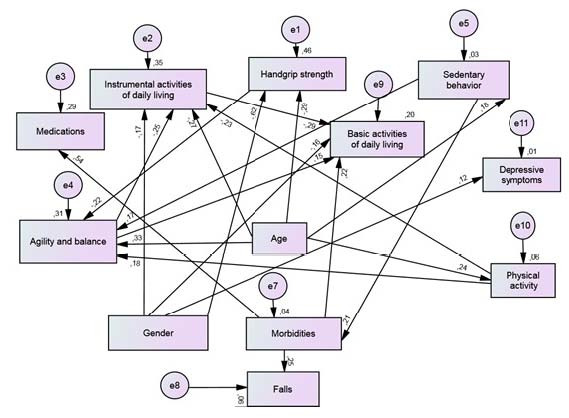
Model used for the analysis of association between sociodemographic, health, and behavioral variables and the occurrence of falls for older adults with abdominal obesity, Alcobaça, Bahia, Brazil, 2021.

**Figure 3.**
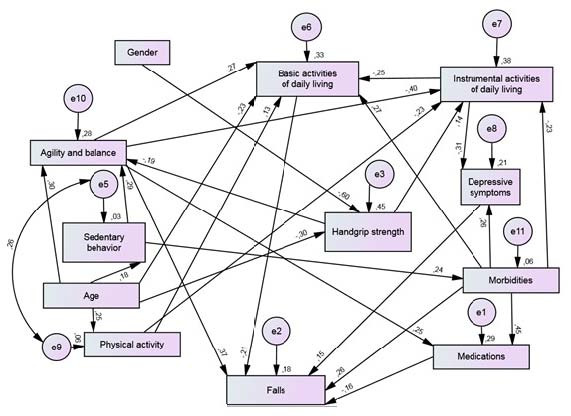
Model for the analysis of the association between sociodemographic, health, and behavioral variables and the occurrence of falls for older adults without abdominal obesity, Alcobaça, Bahia, Brazil, 2021.

Figure 2 shows the association model of sociodemographic, health, and behavioral variables with the occurrence of falls in the last 12 months in participants with abdominal obesity, with the following quality of adjustment indices: (χ^2^ (df = 46) = 66.2, P = 0.03, CFI = 0.97, GFI = 0.96, TLI = 0.95, RMSEA = 0.04). For participants without abdominal obesity, (χ2 (df = 40) = 56.2, P = 0.05, CFI = 0.97, GFI = 0.95, TLI = 0.95, RMSEA = 0.05) was used, and the model is shown in Figure 3.

The direct estimators of the associations between the tested variables and the occurrence of falls according to the presence of abdominal obesity are shown in Table 3. The number of morbidities was directly associated with the incidence of falls in both groups (β = 0.25; P < 0.001 for abdominal obesity and β = 0.26; P = 0.002 without abdominal obesity), suggesting that the frequency of falls in participants increased with the number of morbidities, regardless of obese status (Table 3).

**Table 3.** Direct standardized coefficients for the variables associated with the occurrence of falls among older adults, according to abdominal obesity, Alcobaça, Bahia, Brazil, 2021

Direct effects	With abdominal obesity		Without abdominal obesity	
	Estimator	P^*^	Estimator	P^*^
Morbidities	0.25	< 0.001	0.26	0.002
Agility and dynamic balance	–	–	0.37	< 0.001
Number of depressive symptoms	–	–	0.15	0.039
Number of medications	–	–	- 0.16	0.043
BADL	–	–	-0.21	0.006

* P < 0.05; BADL = basic daily living activities.

Furthermore, in the group of older adults without abdominal obesity, direct associations were also found between a higher incidence of falls and the lowest number of medications (β = -0.16; P = 0.04), the highest number of depressive symptoms (β = 0.15; P = 0.04), worse performance in the agility and dynamic balance tests (β = 0.37; P < 0.001), and lower functional disability for BADL (β = -0.21; P = 0.006) (Table 3).

Regarding indirect mediation of falls, among participants with abdominal obesity, greater exposure to sedentary behavior (β = 0.06), mediated by a greater number of morbidities, was indirectly associated with a greater occurrence of falls (Figure 2).

In participants without abdominal obesity, lower functional disability for BADL mediated an association between advanced age (β = 0.05), lower functional disability for IADL (β = 0.06), and a greater occurrence of falls (Figure 3). Lower handgrip strength (β = -0.07), advanced age (β = 0.11), and greater exposure to sedentary behavior (β = 0.11), mediated by worse performance in the agility and balance test dynamics, were also indirectly associated with a greater number of falls (Figure 3). Furthermore, a greater number of morbidities mediated an indirect association between a greater time spent in sedentary behavior (β = 0.06) and a greater occurrence of falls. A greater number of depressive symptoms also mediated the association between greater functional disability for IADL and the highest number of falls (β = -0.05) (Figure 3).

## DISCUSSION

The prevalence of falls among the participants was 28.6%, similar to that found in previous surveys.^2,5,17^ Regarding the groups, participants with abdominal obesity had a higher prevalence and chance of falling than those without abdominal obesity, in line with a study carried out in older Americans, who were more likely to suffer recurrent falls.^16^ Moreover, a systematic review showed that older adults with obesity were also at higher risk for the occurrence of multiple events other than falls.^18^

Evidence shows that the relationship between obesity and falls can be explained by biomechanics.^18^ The anterior position of the body’s center of mass is assumed in relation to the ankle joint and the need to increase mass to stabilize it on the base of support.^19^ The accumulation of fat in the abdominal region interferes with this postural control mechanism. Thus, the assessment of obesity in the context of preventing falls becomes useful, helping to identify potential risk groups requiring greater interventions.^18^

It should be noted that the use of body mass index (BMI) as the only parameter for the diagnosis of obesity may underestimate the population at risk for falls.^16^ This issue highlights the need to adopt other assessment methods, such as waist circumference, which is easy to measure and use. Moreover, efforts should be made to control obesity and its associated diseases.^34^

Regarding the associations found, morbidities played a direct role in the relationship with falls in both groups. This result among older adults without obesity is similar to that found in other studies.^4,5^ Chronic conditions are prevalent among older adults and are associated with negative outcomes such as years of life lived with disability and can mainly be attributed to low back pain, age-related hearing loss, blindness, oral problems, and diabetes.^35^ Some of these conditions, such as diabetes, cancer, and arterial hypertension, can intensify physical disability if they worsen,^36^ which can predispose to the risk of falls.

In older adults with abdominal obesity, the association between morbidities and falls appears to intensify. Evidence indicates that, in addition to the physiological, physical, sensory, and cognitive changes that occur with the aging process,^1^ the accumulation of adipose tissue in the abdominal region is a risk factor for the development of chronic diseases^34^ and disability in older adults.^20^ These two aspects are also involved in the occurrence of falls.^4,5,8,9,12^ The fact that only the number morbidities was associated with falls in this group suggests that the effect of excess abdominal fat on the expression of morbidities seems to be a more important risk factor than other variables.

Morbidities also mediated indirect associations between sedentary behavior and falls in older adults with and without abdominal obesity. Advanced age increases the propensity to spend more time in sedentary behavior, which in turn causes deleterious effects such as exposure to the risk of chronic diseases.^37^ Abdominal obesity is also a threat to the emergence of these conditions,^34^ which are associated with disability and years of life lost due to premature death.^35^

Although a meta-analysis showed that time spent in sedentary behavior did not increase the chances of being overweight or obese in older adults,^38^ there are greater barriers for older people with these conditions to reducing time in sedentary behavior. These include pre-existing health conditions, feeling of pleasure in activities with lower energy expenditure, environments with adaptation problems, presence of fatigue, and difficulty understanding the differences between sedentary behavior and physical activity.^39^

The direct relationship between the longer time spent performing the agility and dynamic balance test and the occurrence of falls in older adults without abdominal obesity was confirmed by evidence from previous studies.^8,9^ An indirect association also exists between advanced age and falls, mediated by a longer time spent performing the agility and dynamic balance tests. These relationships can be explained by the changes that occur with the aging process in the sensory system of older adults, which can affect balance control and gait pattern, resulting in difficulty adapting to the environment^40^ and performing daily activities.

Still referring to this group, a worse performance in the agility and dynamic balance tests also mediated two other associations: lower handgrip strength and greater exposure to sedentary behavior with the occurrence of falls. Further to advanced age itself being recognized as a factor associated with falls,^2,3,7^ it is known that over the years, changes in body composition occur which are related to a greater occurrence of falls,^7^ including a reduction in muscle mass^20^ and muscle strength^41^. Regarding sedentary behavior, a study carried out in Portuguese older adults showed that a longer time spent in sedentary behavior, regardless of the level of moderate to vigorous physical activity, negatively influences physical fitness components, such as agility and dynamic balance,^42^ which are related to falls.^8,9^

A lower number of medications used in older adults without abdominal obesity was associated with a higher risk of falls in this study sample, which differs from other studies.^10,11^ A study of Spanish older adults showed that 71% of the participants used medications considered to be at risk for falls, such as antidepressants, antipsychotics, sedatives, opioids, and diuretics, suggesting that the type of medication can also influence the risk of falls, in addition to the amount ingested.^10^

Among older adults without abdominal obesity, the highest number of depressive symptoms was positively associated with the occurrence of falls, which corroborates other studies.^3,6^ Clinical symptoms related to depression, such as fear of falling, presence of fatigue, lack of concentration, changes in nutritional status, and gait and balance instability, may predispose to the risk of falls.^43^ Thus, there is a need to integrate the assessment of the risk of falls when older people present with symptoms suggestive of depression.^6^

A higher number of depressive symptoms mediated the association between greater functional disability for IADL and falls. The literature confirms the relationship between functional disability for IADL and depressive symptoms^44,45^ and falls.^12,13^ IADL reflects the ability to socialize and live independently and healthy,^45^ therefore, the inability to perform these activities can cause negative feelings, in addition to reducing the stimulation of physical and cognitive capacity.

The present study also found that the lower the functional disability for BADL, the greater the occurrence of falls in older adults without abdominal obesity. This finding differs from those described in the literature.^12^ It is inferred that the self-perception that older adults have regarding their ability to perform self-care activities, with little or no help from third parties or adaptive equipment, can increase exposure to situations that predispose to falls.

In addition to the aforementioned finding, lower functional disability for BADL mediated the association between lower disability for IADL and older age with a higher occurrence of falls. Regarding the IADL, the relationship can be explained by the existing hierarchy between these activities and the BADL, since the commitment starts with the IADL, and then affects the BADL.^46^ With the human aging process, there is a decline in the sensory system function, which can affect postural stability and displacement,^40^ predisposing to the risk of falls and impairing the performance of ADL.

The strengths of this study include the population type and the path analysis approach, which allows the investigation of direct and indirect associations between variables and can help identify risk groups and target more specific interventions. Among the limitations of the study are the cross-sectional design, which did not allow determination of a causal relationship; the use of subjective and self-reported measures such as BADL and IADL, sedentary behavior, and physical activity practice; and the limited population studied, which was composed only of older people registered in the municipality’s Family Health Strategy.

## CONCLUSION

A high number of morbidities was the only factor directly associated with falls in older adults with abdominal obesity. Conversely, in the group without abdominal obesity, besides the higher number of morbidities, an association was identified between falls and a higher number of medications, a higher number of depressive symptoms, a longer time to perform the agility and dynamic balance tests, and less functional disability for BADL. This set of findings can help to understand the complexity of factors associated with falls in older adults and allows the identification of individuals at greater risk for falls. In our study, this group was older adults with abdominal obesity.
